# Supplementary material for: 5-Fluorouracil treatment induces characteristic T>G mutations in human cancer
Source: Nat Commun. 2019 Oct 8;10:4571. doi: 10.1038/s41467-019-12594-8 (PMC6783534; doi:10.1038/s41467-019-12594-8)
Supplement: Supplementary file 1 — Supplementary Information [file 41467_2019_12594_MOESM1_ESM.pdf]

Supplementary Information

5-Fluorouracil treatment induces characteristic T>G mutations in human cancer

Sharon Christensen, Bastiaan Van der Roest, Nicolle Besselink, Roel Janssen, Sander Boymans, John W.M. Martens, Marie-Laure Yaspo, Peter Priestley, Ewart Kuijk, Edwin Cuppen, Arne Van Hoeck

Supplementary Tables

Supplementary Table 1. **Comparison of *de novo* signatures with COSMIC signatures**

Signature_denovo	COSMIC	Cosine similarity	Alexandrov 2018	Cosine similarity	Boot 2018	Cosine similarity
NMF_A		NA	SBS41	0.71	Ant	0.9
NMF_B	Signature.6	0.76	SBS44	0.87		
NMF_C	Signature.15	0.81	SBS15	0.71		
NMF_D		NA	SBS34	0.62		
NMF_E	Signature.1	0.97	SBS1	0.97		
NMF_F	Signature.18	0.93	SBS18	0.96		
NMF_G	Signature.3	0.9	SBS37	0.89		
NMF_H	Signature.17	0.97	SBS17b	0.97		
NMF_I	Signature.8	0.78	SBS8	0.81		
NMF_J	Signature.13	1	SBS13	0.95		
NMF_K	Signature.2	1	SBS2	1		
NMF_L	Signature.20	0.77	SBS44	0.87		
NMF_M	Signature.7	0.97	SBS7a	0.98		
NMF_N	Signature.10	0.92	SBS10a	0.78		
NMF_O	Signature.5	0.51	SBS5	0.51		
NMF_P	Signature.29	0.82	SBS29	0.82		

De novo signatures are compared with the highest cosine similarity to the i) COSMIC signatures (first column), ii) signatures as reported in Alexandrov et al.[1], (second column) and iii) signature as reported in Boot et al.,[2] (last column).

Supplementary Table 2. **Subclonal driver mutations caused by the 5-FU mutational process**

Cohort	Chromosome	Position	Ref	Alt	Gene	Clonality	Trinucl_ context	Type	Canonical	Ref_ original	Alt_ original	Moa	Driver
5-FU pretreated colon	Chr 12	25380275	T	G	*KRAS*	SUBCLONAL	CTT	SNP	Missense variant	T	G	Act	Yes
5-FU pretreated colon	Chr 3	178936091	C	T	*PIK3CA*	SUBCLONAL	TCA	SNP	Missense variant	G	A	Act	Yes
5-FU pretreated colon	Chr 3	178936095	T	G	*PIK3CA*	SUBCLONAL	CTG	SNP	Missense variant	A	C	Act	Yes
5-FU pretreated colon	Chr 4	153247366	C	T	*FBXW7*	SUBCLONAL	TCG	SNP	Missense variant	C	T	LoF	Yes
5-FU pretreated colon	Chr 5	11273704	C	T	*APC*	SUBCLONAL	TCG	SNP	Stop gained	C	T	LoF	Yes
Not 5-FU pretreated colon	Chr 17	7577539	C	T	*TP53*	SUBCLONAL	CCG	SNP	Missense variant	G	A	LoF	Yes
Not 5-FU pretreated colon	Chr 4	153249385	C	T	*FBXW7*	SUBCLONAL	GCG	SNP	Missense variant	G	A	LoF	Yes
5-FU pretreated breast	Chr 3	138417865	C	T	*PIK3CB*	SUBCLONAL	TCA	SNP	Missense variant	C	T	Act	Yes
5-FU pretreated breast	Chr 3	178936082	C	T	*PIK3CA*	SUBCLONAL	TCA	SNP	Missense variant	G	A	Act	Yes
5-FU pretreated breast	Chr 3	178952085	T	C	*PIK3CA*	SUBCLONAL	ATG	SNP	Missense variant	A	G	Act	Yes
5-FU pretreated breast	Chr 6	152419920	T	G	*ESR1*	SUBCLONAL	CTC	SNP	Missense variant	T	G	Act	Yes
5-FU pretreated breast	Chr 6	152419926	T	C	*ESR1*	SUBCLONAL	GTC	SNP	Missense variant	A	G	Act	Yes
Not 5-FU pretreated breast	Chr 10	89711893	C	T	*PTEN*	SUBCLONAL	TCA	SNP	Stop gained	C	T	LoF	Yes
Not 5-FU pretreated breast	Chr 3	178916891	C	T	*PIK3CA*	SUBCLONAL	CCG	SNP	Missense variant	G	A	Act	Yes
Not 5-FU pretreated breast	Chr 3	17896091	C	T	*PIK3CA*	SUBCLONAL	TCA	SNP	Missense variant	G	A	Act	Yes
Not 5-FU pretreated breast	Chr 6	152419923	T	G	*ESR1*	SUBCLONAL	ATA	SNP	Missense variant	A	C	Act	Yes
Not 5-FU pretreated breast	Chr 6	152419926	T	C	*ESR1*	SUBCLONAL	GTC	SNP	Missense variant	A	G	Act	Yes

Patient cancer type and 5-FU treatment status are indicated in the first column, followed by the chromosomal position (columns 2 and 3), the mutation (with reference in column 4 and mutation in column 5) and cancer driver gene (column 6). The clonality, trinucleotide context and mutation type are summarized in columns 7-14.

Supplementary Figures

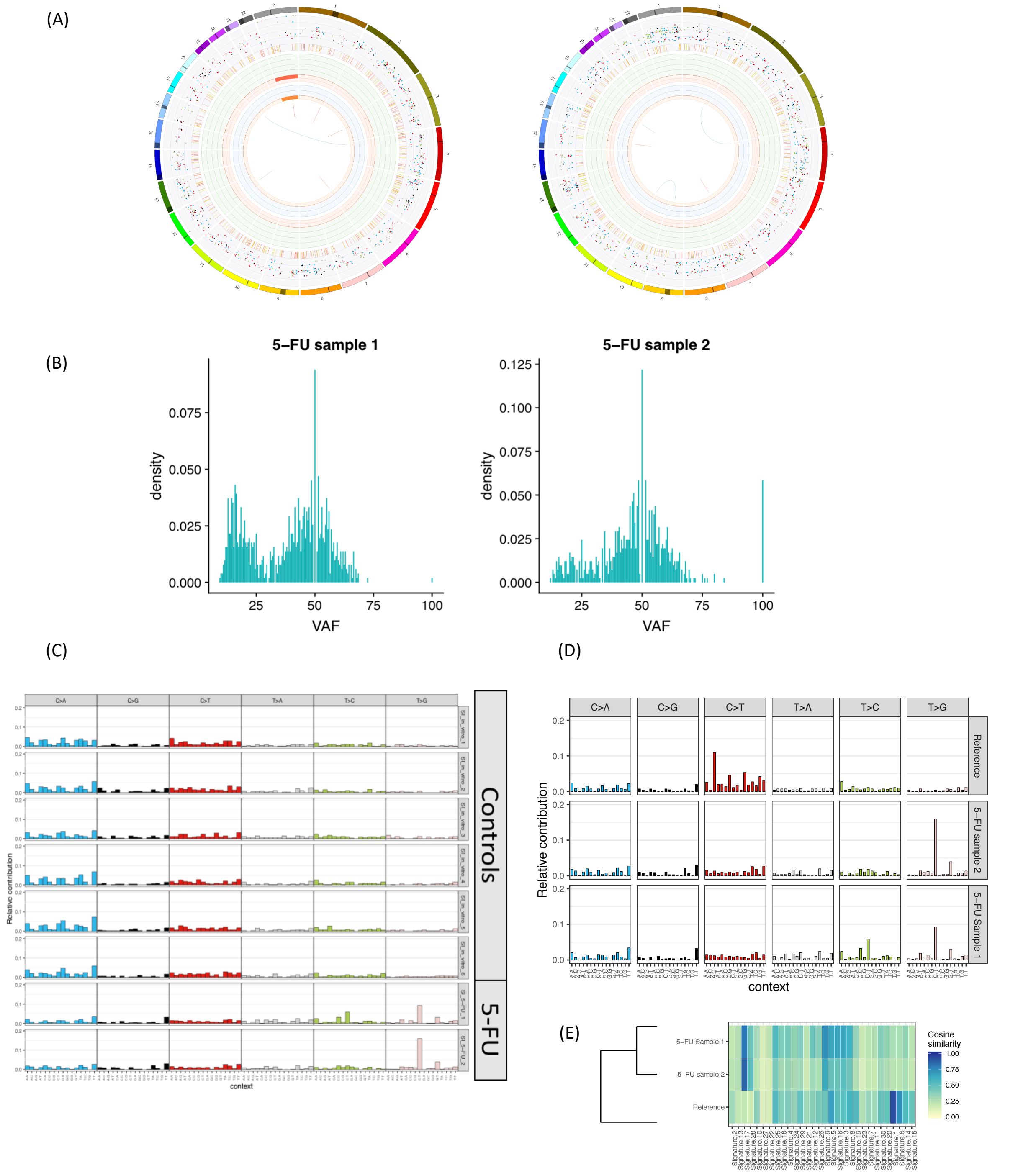

Supplementary Figure 1. **Analysis of mutations in 5-FU treated organoids.** (A) Circos plots showing the somatic events of the two organoid lines treated with 5-FU. The outer first circle shows the chromosomes. The second circle shows the somatic variants (incl. exon, intron and intergenic regions). Somatic variants are further divided into an outer ring of SBSs and an inner ring of INDELs. Each dot represents a single somatic variant scaled from to 100% by its allele frequency

score. SBSs are colored according to the type of base change (e.g. C>T/G>A in red) and are in concordance with the coloring used in Alexandrov et al. 2013 Nature paper that describes the use of mutational signatures[3]. INDELs are colored yellow and red for insertions and deletions respectively. The third circle shows all observed tumor purity adjusted copy number changes. Copy number losses are indicated in red, green shows regions of copy number gain. The scale ranges from 0 (complete loss) to 6 (high level gains). If the absolute copy number is > 6 it is shown as 6 with a green dot on the diagram. The fourth circle represents the observed 'minor allele copy numbers' across the chromosome. The range of the chart is from 0 to 3. The expected normal minor allele copy number is 1, and anything below 1 is shown as a loss (orange) and represents a LOH event. Minor allele copy numbers above 1 (blue) indicate amplification events of both A and B alleles at the indicated locations. The innermost circle displays the observed structural variants within or between the chromosomes. Translocations are indicated in blue, deletions in red, insertions in yellow, tandem duplications in green and inversions in black. (B) Histogram showing the SBS allele frequencies of all SBSs. SBSs with a VAF score between 0.3 and 0.7 are considered as clonal which were used for *de novo* mutational pattern characterization. (C) Mutational spectra of all SBSs for each human intestinal organoid line used for 5-FU mutational pattern characterization. The six upper samples are non-exposed control organoid lines, while the two samples below are 5-FU exposed organoids. Different mutation types and the direct sequence context are indicated. (D) Mutational spectra of all SBSs for the isogenic organoid line (using matching blood DNA as reference) and the 5-FU exposed organoid lines. (E) Heat map showing the cosine similarity scores for each indicated sample and all COSMIC signatures.

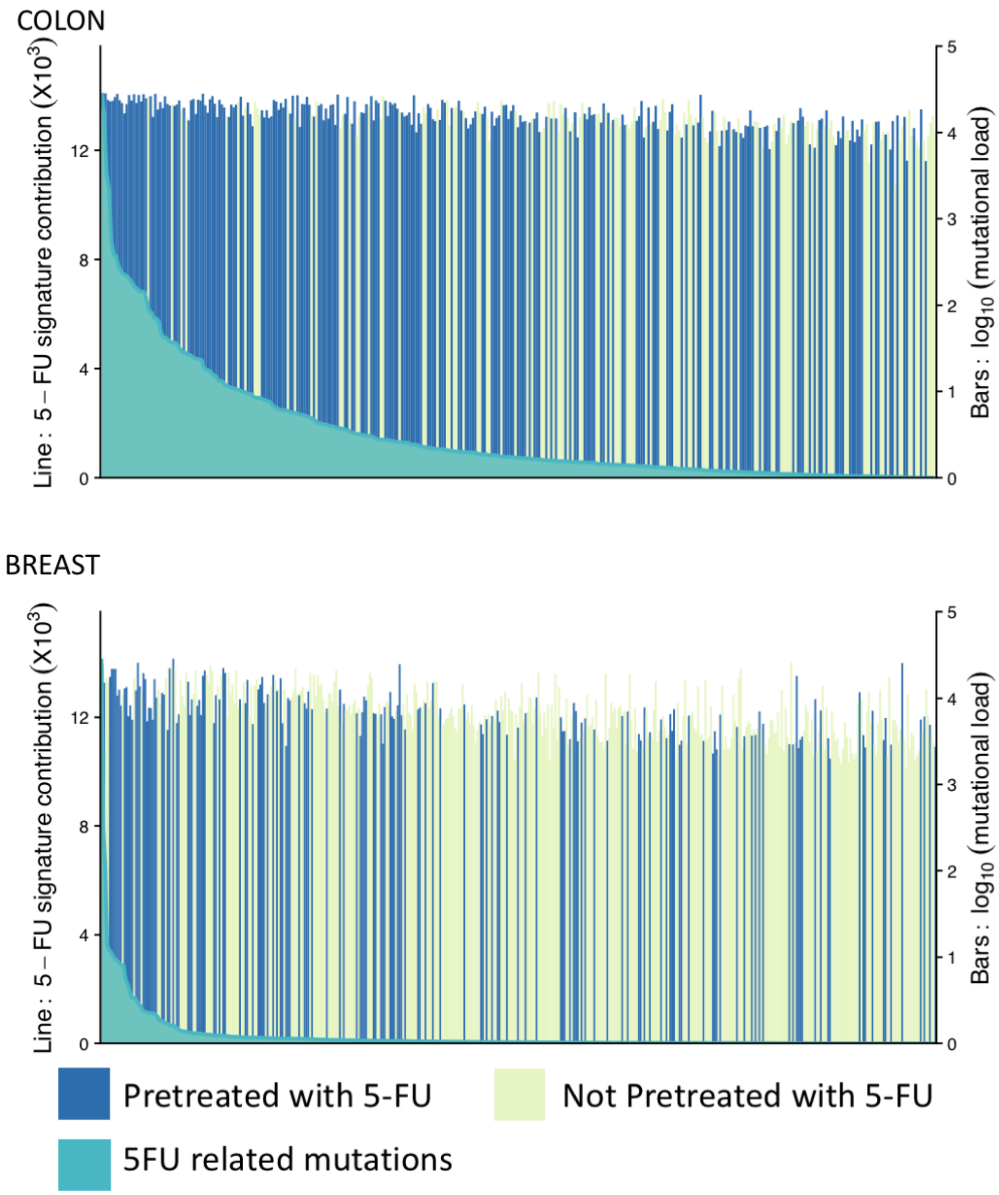

Supplementary Figure 2. **5-FU signature contributions in colon and breast cancer cohort.** Mutational distribution plot of each sample for the colon (upper) and breast (lower) cancer cohort. The green bars show the absolute mutation contribution of the 5-FU signature whereas the blue (5-FU pretreated patients) and yellow (not 5-FU pretreated patients) bars illustrate the overall tumor mutational burden for each sample. The samples have been ordered according to their 5-FU signature contribution.

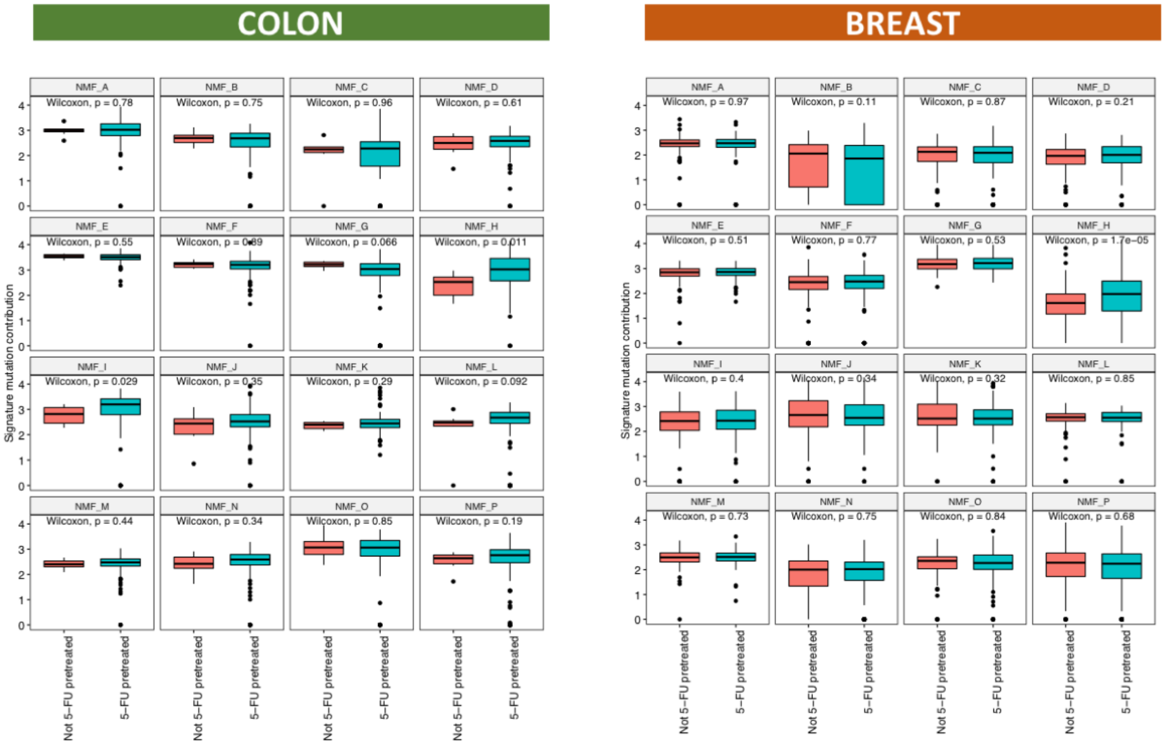

Supplementary Figure 3. **Absolute contributions of *de novo* signatures extracted using NMF.** Absolute mutational contribution for each *de novo* signature in the colon (left) and breast (right) cancer cohort. NMF_H, the only signature significantly increased upon 5-FU treatment in both cohorts, resembles the 5-FU *in-vitro* mutation spectrum and is assigned to "5-FU signature" in the main text. All box-and whiskers plots display the first and the third quartiles (top and bottom of the box), the median (vertical line inside the box), the extremes (whiskers) and, if present, the outliers (single dots).

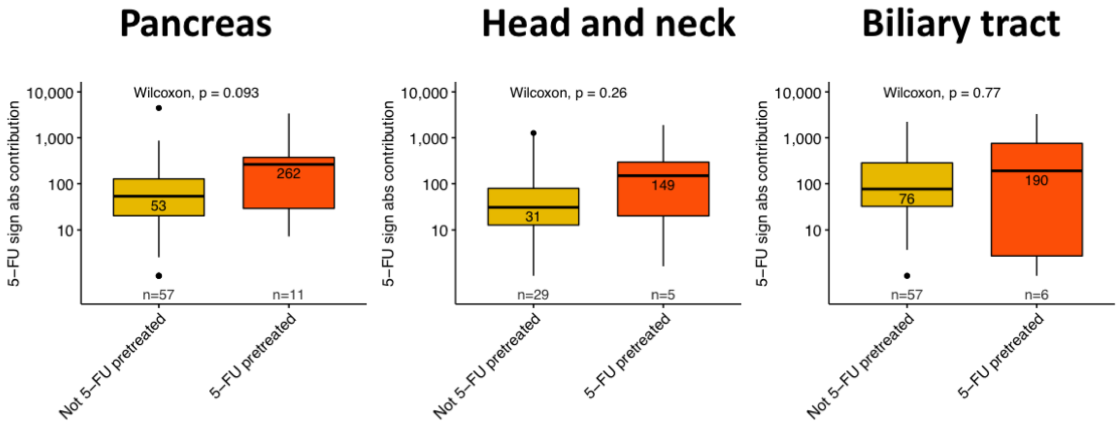

Supplementary Figure 4. **Comparison of 5-FU and capecitabine treated patients with untreated patients.** Box- and whisker plots indicating the absolute contribution for the 5-FU obtained *de novo* pattern between 5-FU pretreated and not 5-FU pretreated cancer patients for different cancer types. A Wilcoxon rank-sum test between every cohort was performed and each p-value is illustrated at the top of the plots. All box-and whiskers plots display the first and the third quartiles (top and bottom of the box), the median (vertical line inside the box), the extremes (whiskers) and, if present, the outliers (single dots).

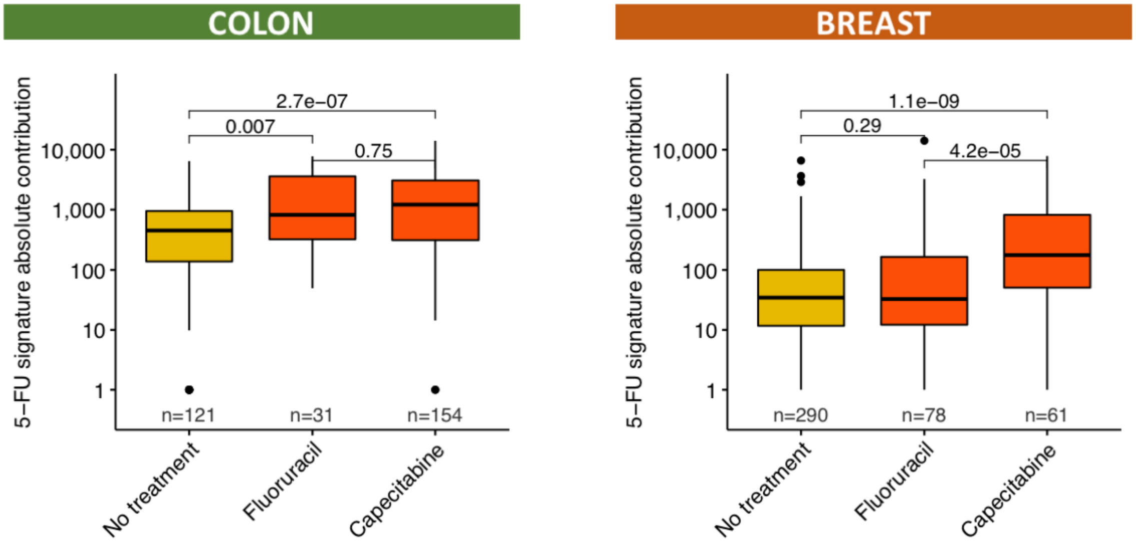

Supplementary Figure 5. **Comparison of 5-FU and capecitabine treated patients with untreated patients.** Box- and whisker plot indicating the absolute contribution for the 5-FU obtained *de novo* pattern between patients pretreated with fluorouracil or capecitabine and not pretreated cancer patients. A Wilcoxon rank-sum test between every cohort was performed and each p-value is illustrated at the top of the plots. All box-and whiskers plots display the first and the third quartiles (top and bottom of the box), the median (vertical line inside the box), the extremes (whiskers) and, if present, the outliers (single dots).

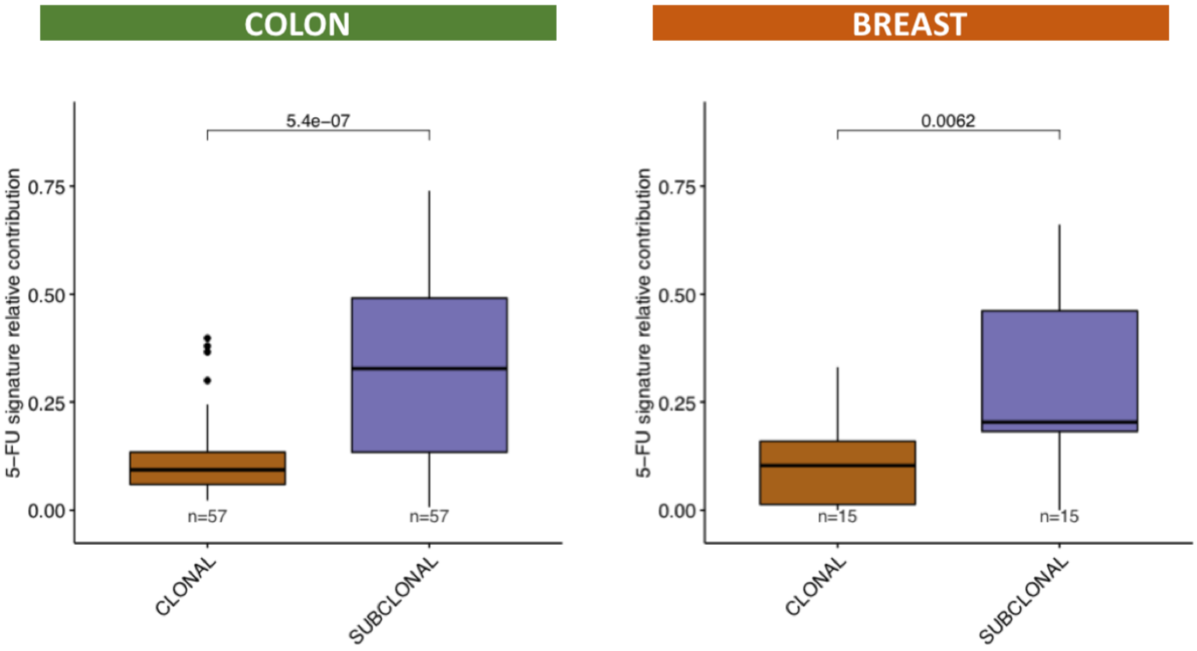

Supplementary Figure 6. **Clonal and subclonal analysis for 5-FU mutations.** Box-and whisker plot indicating the relative contribution of 5-FU signature between clonal and subclonal mutations from 5-FU pretreated cancer patients. The left plot is obtained from the colon cancer cohort while the plot illustrated on the right is obtained from breast cancer cohort. A Wilcoxon rank-sum test between every cohort was performed and each p-value is illustrated at the top of the plots. All box-and whiskers plots display the first and the third quartiles (top and bottom of the box), the median (vertical line inside the box), the extremes (whiskers) and, if present, the outliers (single dots).

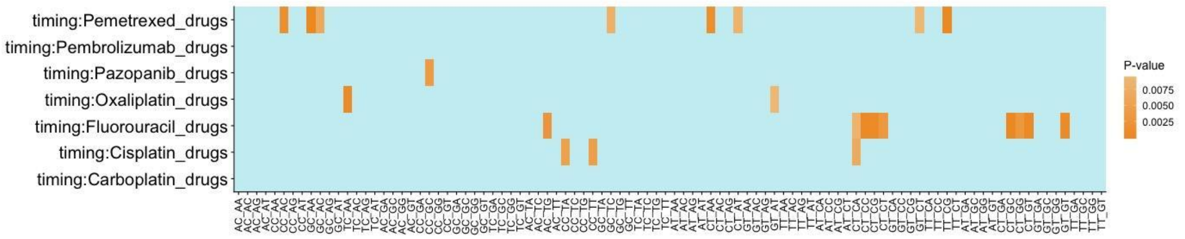

Supplementary Figure 7. **Significance of mutations in patients treated with several drugs between biopsies.** Heatmap showing the p-values of the linear mixed model regression analysis on the normalized mutation counts of each mutation type (x-axis) and each treatment type (y-axis) between patients who received 5-FU treatment between the two biopsies and patients not treated with 5-FU between two biopsies. In the model, we controlled for exposure dose and time as well as other therapies that were administered to the patient between the first and second biopsy. P-values were obtained by performing an ANOVA test on the regression model.

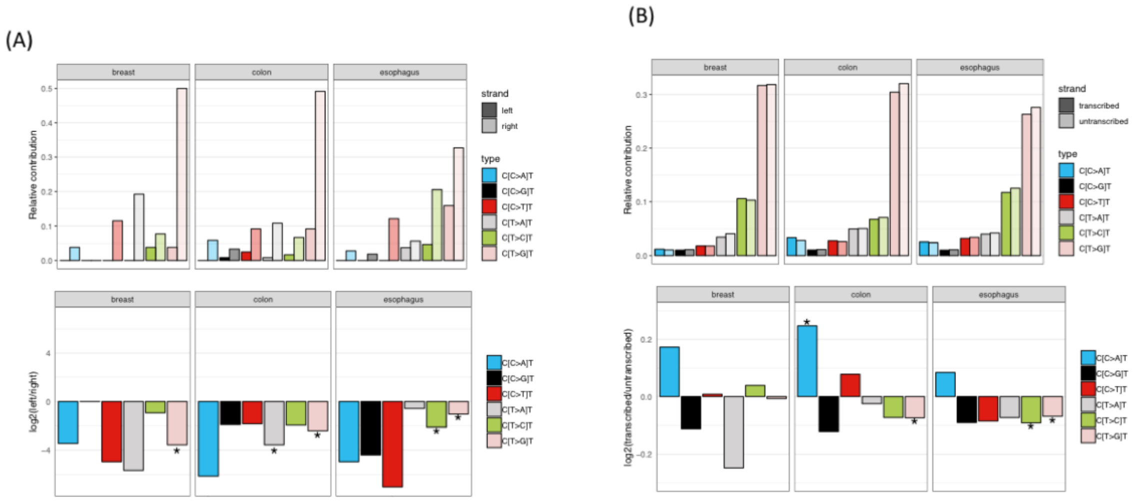

Supplementary Figure 8. **Comparison of signature 17-like signatures in 5-FU treated and untreated cancers.** (A) Replication strand bias of C[N>N]T mutations in 5-FU pretreated colon and breast samples and not 5-FU pretreated esophagus samples. Relative levels of each base substitution type in the left (leading) and right (lagging) DNA strands are shown for each cohort. The log2 ratio of the number of SBSs on the left and right strand shows the effect size. Asterisks indicate a significant difference (P < 0.05, two-sided Poisson test). (B) Transcription strand bias of C[N>N]T mutations in 5-FU pretreated colon and breast samples and not 5-FU pretreated esophagus samples. Relative levels of each base substitution type in the transcribed and untranscribed DNA strands are shown for each cohort. The log2 ratio of the number of the number of SBSs on the transcribed and untranscribed strand shows the effect size. Asterisks indicate a significant difference (P < 0.05, two-sided Poisson test).

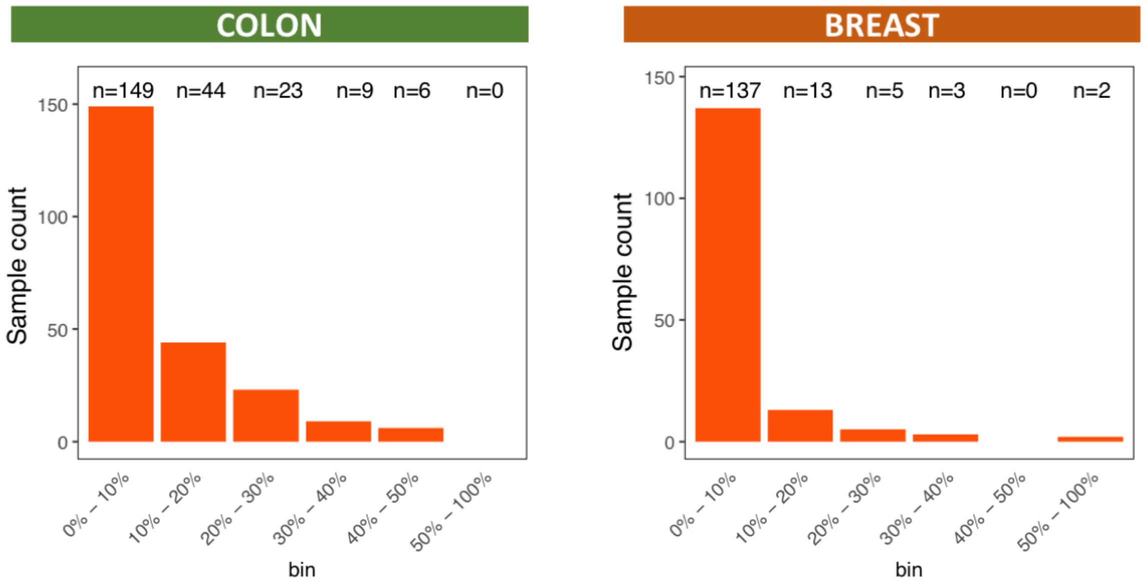

Supplementary Figure 9. **Impact of 5-FU treatment on the tumor mutational burden.** Histograms illustrating the relative 5-FU contribution to its respectively tumor mutational burden for all the colon (left) and breast (right) cancer patients.

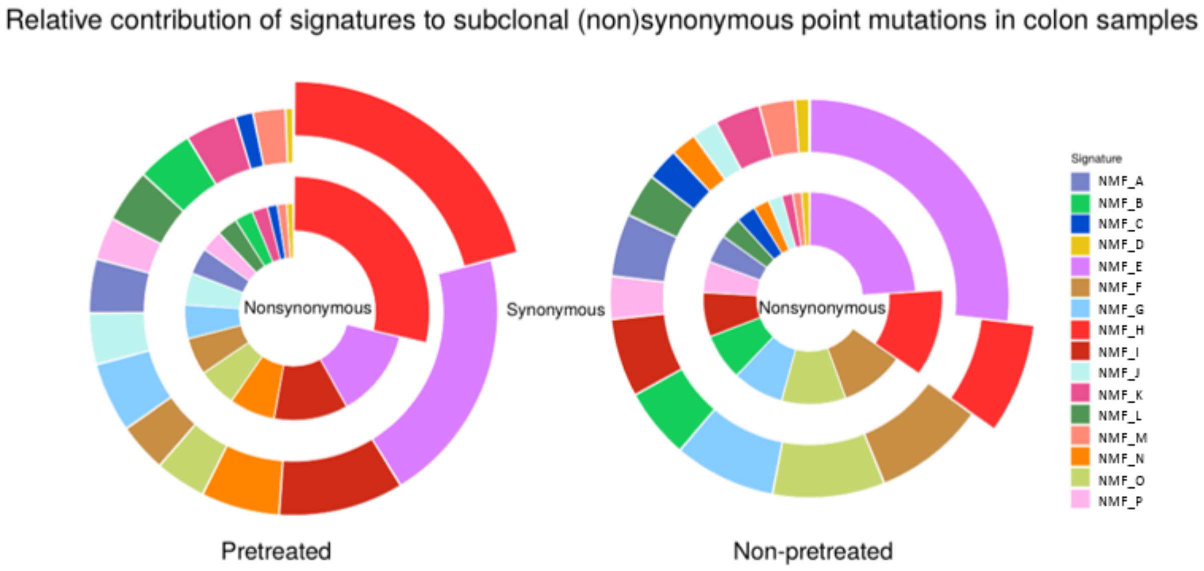

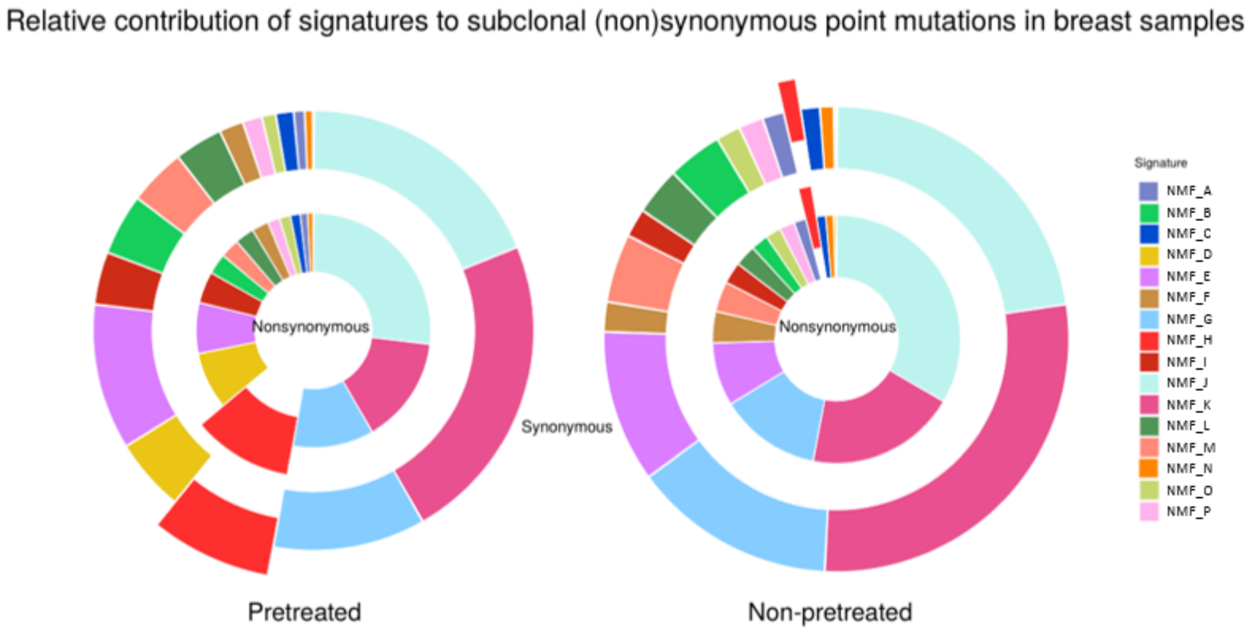

Supplementary Figure 10. **Distribution of the SBSs associated with mutational patterns.** The probability of each SBS being due to each mutational process was estimated. The probabilities over all the SBSs were summed per mutational pattern to obtain the cumulative probabilities across all mutational patterns illustrated in pie charts. NMF_H was identified as 5-FU signature. The selected mutations, which are considered to be induced by 5-FU treatment, are used for dN/dS analysis.

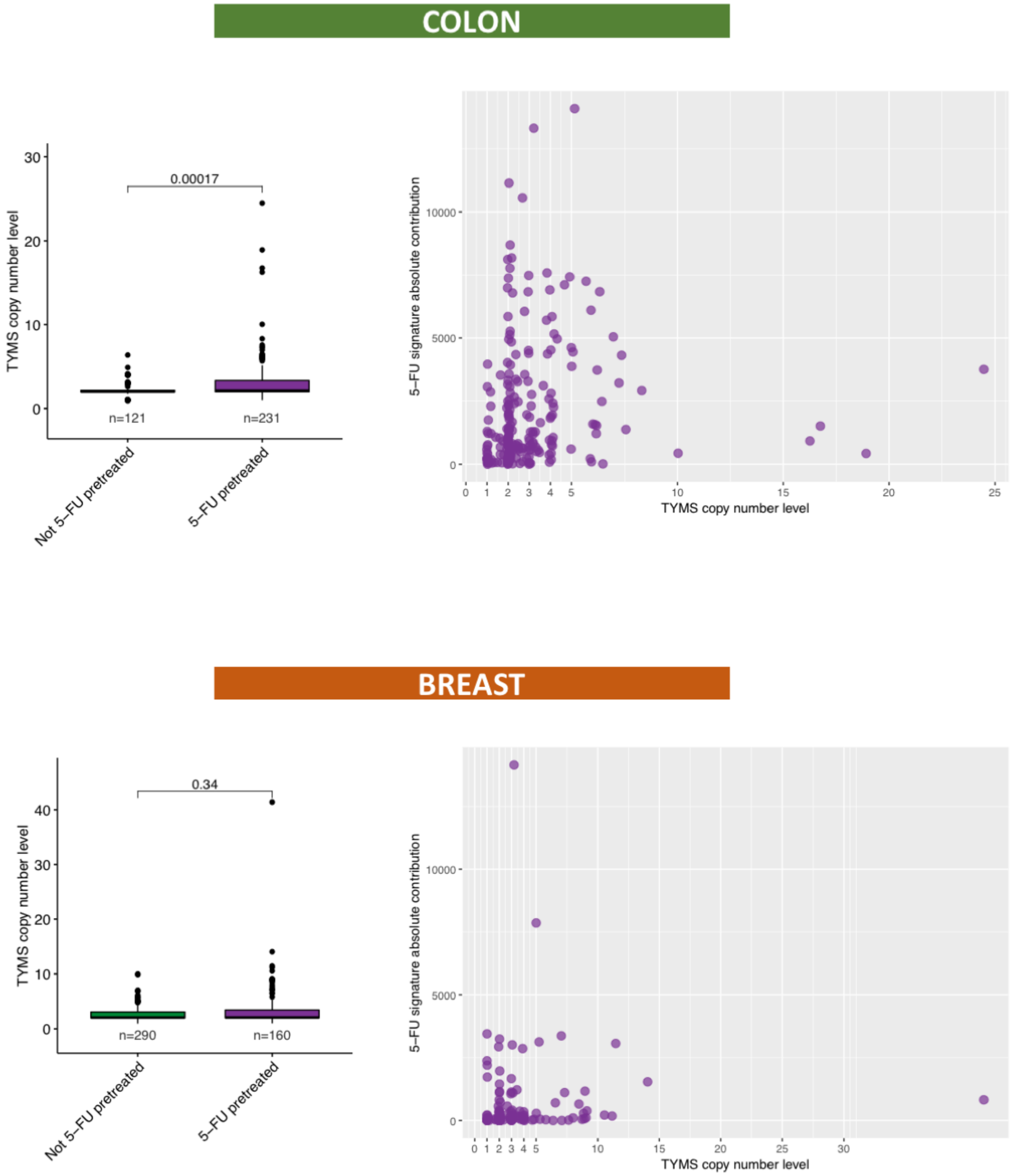

Supplementary Figure 11. **Analyses of TYMS copy number related to 5-FU treatment and NMF_H.** (Left) Box- and whisker plot indicating the TYMS copy number levels between 5-FU pretreated and not 5-FU pretreated cancer patients. A Wilcoxon rank-sum test between every cohort was performed and each p-value is illustrated at the top of the plots. (Right) Scatterplot showing the TYMS copy number levels to the absolute contribution of NMF_5FU. All box-and whiskers plots display the first and the third quartiles (top and bottom of the box), the median (vertical line inside the box), the extremes (whiskers) and, if present, the outliers (single dots).

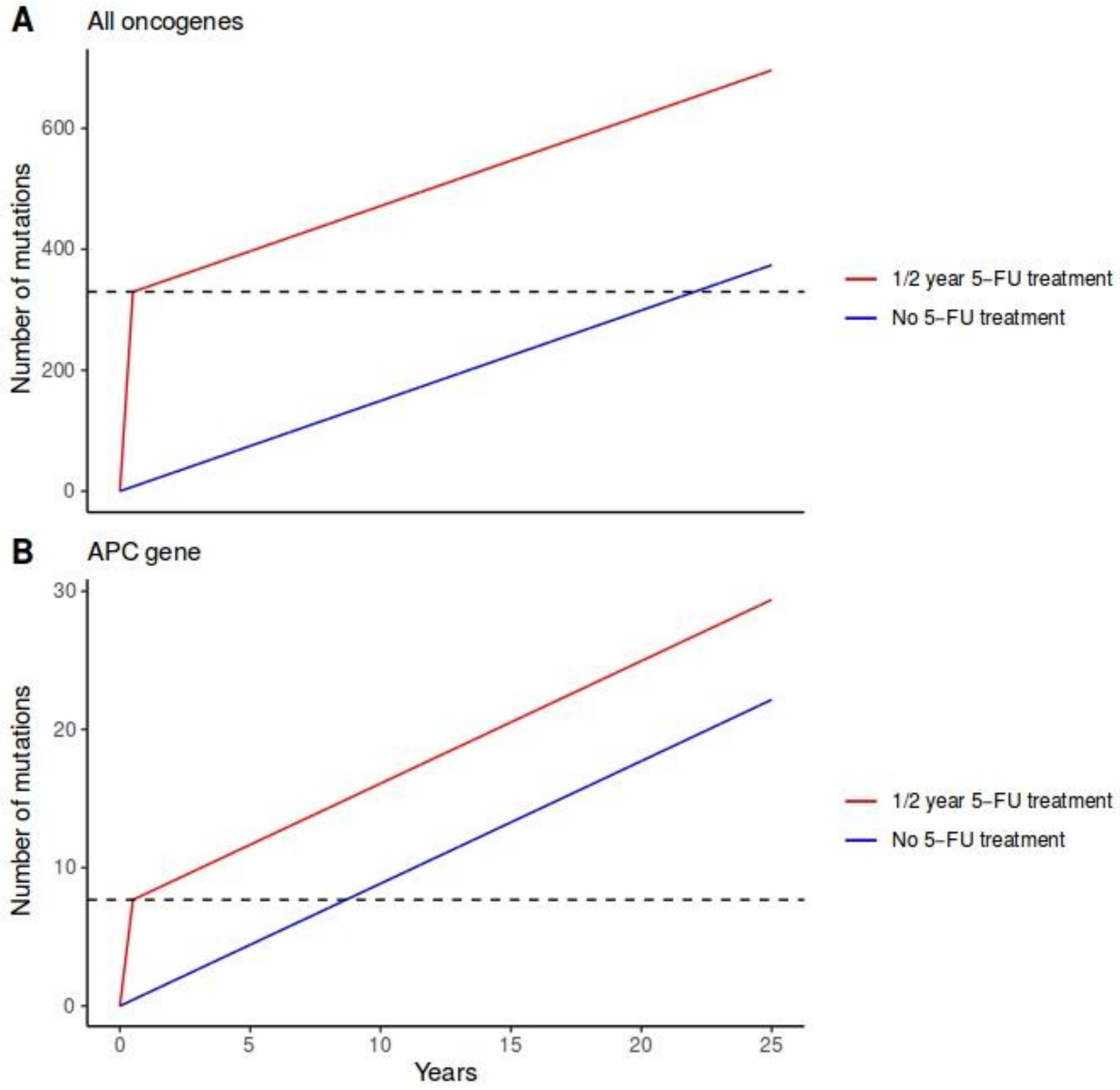

Supplementary Figure 12. **Occurrences of oncogenic mutations after half a year of 5-FU treatment.** (A)
Oncogenic mutation accumulation in colon stem cells of one full cycle of 5-FU treatment (red, half a year treatment [until dashed line] and subsequent 'normal' oncogenic mutation accumulation) versus 'normal' oncogenic mutation accumulation (blue). Half a year treatment of mutation accumulation is worth 20 years of 'normal' mutation accumulation, represented by the dashed line. (B) Oncogenic mutation accumulation in *APC* of one full cycle of 5-FU treatment (red, half a year treatment [until dashed line] and subsequent 'normal' oncogenic mutation accumulation) reflects 'normal' oncogenic mutation accumulation (blue) in this gene of colon stem cells of about 10 years.

Supplementary References

1. Alexandrov, L. B. *et al.* The Repertoire of Mutational Signatures in Human Cancer. *bioRxiv* 322859 (2018). doi:10.1101/322859
2. Boot, A. *et al.* Mutational signature analysis of Asian OSCCs reveals novel mutational signature with exceptional sequence context specificity. (2018). doi:10.1101/368753
3. Alexandrov, L. B. *et al.* Signatures of mutational processes in human cancer. *Nature* **500**, 415–421 (2013).
