# Supplementary material for: 5-Fluorouracil treatment induces characteristic T>G mutations in human cancer
Source: Nat Commun. 2019 Oct 8;10:4571. doi: 10.1038/s41467-019-12594-8 (PMC6783534; doi:10.1038/s41467-019-12594-8)
Supplement: Supplementary file 3 — Reporting Summary [file 41467_2019_12594_MOESM3_ESM.pdf]

nature research

Corresponding author(s): Edwin Cuppen

Last updated by author(s): Jul 31, 2019

Reporting Summary

Nature Research wishes to improve the reproducibility of the work that we publish. This form provides structure for consistency and transparency in reporting. For further information on Nature Research policies, see Authors & Referees and the Editorial Policy Checklist.

Statistics

For all statistical analyses, confirm that the following items are present in the figure legend, table legend, main text, or Methods section.

n/a	Confirmed	
☐	☒	The exact sample size (*n*) for each experimental group/condition, given as a discrete number and unit of measurement
☐	☒	A statement on whether measurements were taken from distinct samples or whether the same sample was measured repeatedly
☐	☒	The statistical test(s) used AND whether they are one- or two-sided<br>*Only common tests should be described solely by name; describe more complex techniques in the Methods section.*
☒	☐	A description of all covariates tested
☐	☒	A description of any assumptions or corrections, such as tests of normality and adjustment for multiple comparisons
☒	☐	A full description of the statistical parameters including central tendency (e.g. means) or other basic estimates (e.g. regression coefficient) AND variation (e.g. standard deviation) or associated estimates of uncertainty (e.g. confidence intervals)
☒	☐	For null hypothesis testing, the test statistic (e.g. *F*, *t*, *r*) with confidence intervals, effect sizes, degrees of freedom and *P* value noted<br>*Give P values as exact values whenever suitable.*
☒	☐	For Bayesian analysis, information on the choice of priors and Markov chain Monte Carlo settings
☒	☐	For hierarchical and complex designs, identification of the appropriate level for tests and full reporting of outcomes
☐	☒	Estimates of effect sizes (e.g. Cohen's *d*, Pearson's *r*), indicating how they were calculated

Our web collection on statistics for biologists contains articles on many of the points above.

Software and code

Policy information about availability of computer code

Data collection	WGS data and corresponding clinical data have been obtained from external sources :<br>Metastatic cancer data was obtained from Hartwig Medical Foundation and provided under data request number DR-047. Both WGS and clinical data is freely available for academic use from the Hartwig Medical Foundation through standardized procedures and request forms can be found at https://www.hartwigmedicalfoundation.nl.<br>Primary breast cancer data was downloaded from the ICGC data portal on August 2, 2017.<br>Primary colon cancer samples were kindly shared by Max-Planck-Institute with a signed agreement for data and sample transfer.<br>RepliSeq data was downloaded from https://bitbucket.org/bsblabludwig/replicationasymmetry/src/master/.
Data analysis	All code used for data analysis and code used is publicly available and can be found at https://github.com/UMCUGenetics/5FU. We used software from the following external resources: https://github.com/hartwigmedical/pipeline, https://github.com/hartwigmedical/hmftools, https://github.com/UMCUGenetics/MutationalPatterns/, https://github.com/im3sanger/dndscv

For manuscripts utilizing custom algorithms or software that are central to the research but not yet described in published literature, software must be made available to editors/reviewers. We strongly encourage code deposition in a community repository (e.g. GitHub). See the Nature Research guidelines for submitting code & software for further information.

Data

Policy information about availability of data

All manuscripts must include a data availability statement. This statement should provide the following information, where applicable:

- Accession codes, unique identifiers, or web links for publicly available datasets
- A list of figures that have associated raw data
- A description of any restrictions on data availability

WGS data and corresponding clinical data have been obtained from the Hartwig Medical Foundation and provided under data request number DR-047. Both WGS and clinical data is freely available for academic use from the Hartwig Medical Foundation through standardized procedures and request forms can be found at https://www.hartwigmedicalfoundation.nl. The human sequencing data of the 5-FU treated and control organoid lines have been deposited at the European

Genome-phenome Archive (http://www.ebi.ac.uk/ega/) under accession numbers EGAS00001003592 and EGAS00001002955, respectively. Data access for both EGA data repositories is controlled by the Division of Biomedical Genetics UMC Utrecht Data Access Committee (DAC).

Field-specific reporting

Please select the one below that is the best fit for your research. If you are not sure, read the appropriate sections before making your selection.

☒ Life sciences ☐ Behavioural & social sciences ☐ Ecological, evolutionary & environmental sciences

For a reference copy of the document with all sections, see nature.com/documents/nr-reporting-summary-flat.pdf

Life sciences study design

All studies must disclose on these points even when the disclosure is negative.

Sample size	For In vitro analysis, we used all the clonal somatic mutations (N= 1,324) to characterize the 5FU specific mutation profile. The somatic mutations were characterized from 2 independent in vitro experiments.<br>For In vivo analysis, the sample size of all statistical tests was determined based on the reported 5-FU treatment and pretreatment status.
Data exclusions	Human WGS samples for which pretreatment was not documented (hasSystemicPreTreatment = NA) were excluded from this study.
Replication	2 independent human small intestinal organoid cultures exposed to 5-FU were set up to serve as both technical and biological replicates. (This information will be better specified in the material and methods section during revision process.)
Randomization	Not relevant for this study.
Blinding	NA

Reporting for specific materials, systems and methods

We require information from authors about some types of materials, experimental systems and methods used in many studies. Here, indicate whether each material, system or method listed is relevant to your study. If you are not sure if a list item applies to your research, read the appropriate section before selecting a response.

Materials & experimental systems

n/a	Involved in the study	
☒	☐	Antibodies
☐	☒	Eukaryotic cell lines
☒	☐	Palaeontology
☒	☐	Animals and other organisms
☐	☒	Human research participants
☒	☐	Clinical data

Methods

n/a	Involved in the study	
☒	☐	ChIP-seq
☒	☐	Flow cytometry
☒	☐	MRI-based neuroimaging

Eukaryotic cell lines

Policy information about cell lines

Cell line source(s)	The healthy human small intestinal organoid line was obtained from UMC Utrecht with the approval for using of this material for research purposes following STEM protocol.
Authentication	The human small intestinal organoid lines were whole genome sequenced to confirm identity.
Mycoplasma contamination	The organoid lines were negatively tested for mycoplasma contamination.
Commonly misidentified lines (See ICLAC register)	NA

Human research participants

Policy information about studies involving human research participants

Population characteristics	Small intestinal biopsy samples were obtained from an individual that had been admitted for suspected inflammation. The individual was found to be healthy based on standard histological examination. Endoscopic biopsies were performed at the University Medical Center Utrecht.

Recruitment	Small intestinal biopsy samples were obtained from an individual that had been admitted for suspected inflammation. The individual was found to be healthy based on standard histological examination. Endoscopic biopsies were performed at the University Medical Center Utrecht.
Ethics oversight	The patients' informed consent was obtained and this study was approved by the ethical committee of University Medical Center Utrecht.

Note that full information on the approval of the study protocol must also be provided in the manuscript.
